# Biochemical Characterization of the *GBA2* c.1780G>C Missense Mutation in Lymphoblastoid Cells from Patients with Spastic Ataxia

**DOI:** 10.3390/ijms19103099

**Published:** 2018-10-10

**Authors:** Anna Malekkou, Maura Samarani, Anthi Drousiotou, Christina Votsi, Sandro Sonnino, Marios Pantzaris, Elena Chiricozzi, Eleni Zamba-Papanicolaou, Massimo Aureli, Nicoletta Loberto, Kyproula Christodoulou

**Affiliations:** 1 Biochemical Genetics Department, The Cyprus Institute of Neurology and Genetics, Nicosia 1683, Cyprus; annama@cing.ac.cy; 2 Cyprus School of Molecular Medicine, Nicosia 1683, Cyprus; votsi@cing.ac.cy (C.V.); pantzari@cing.ac.cy (M.P.); ezamba@cing.ac.cy (E.Z.-P.); roula@cing.ac.cy (K.C.); 3 Department of Medical Biotechnology and Translational Medicine, University of Milano, 20122 Milano, Italy; maura.samarani@unimi.it (M.S.); sandro.sonnino@unimi.it (S.S.); elena.chiricozzi@unimi.it (E.C.); nicoletta.loberto@unimi.it (N.L.); 4 Neurogenetics Department, The Cyprus Institute of Neurology and Genetics, Nicosia 1683, Cyprus; 5 Neurology Clinic C, The Cyprus Institute of Neurology and Genetics, Nicosia 1683, Cyprus; 6 Neurology Clinic D, The Cyprus Institute of Neurology and Genetics, Nicosia 1683, Cyprus

**Keywords:** GBA2, non-lysosomal β-glucosylceramidase, β-glucocerebrosidase, spastic ataxia, glucosylceramide, plasma membrane, lymphoblastoid cell lines

## Abstract

The *GBA2* gene encodes the non-lysosomal glucosylceramidase (NLGase), an enzyme that catalyzes the conversion of glucosylceramide (GlcCer) to ceramide and glucose. Mutations in *GBA2* have been associated with the development of neurological disorders such as autosomal recessive cerebellar ataxia, hereditary spastic paraplegia, and Marinesco-Sjogren-Like Syndrome. Our group has previously identified the *GBA2* c.1780G>C [p.Asp594His] missense mutation, in a Cypriot consanguineous family with spastic ataxia. In this study, we carried out a biochemical characterization of lymphoblastoid cell lines (LCLs) derived from three patients of this family. We found that the mutation strongly reduce NLGase activity both intracellularly and at the plasma membrane level. Additionally, we observed a two-fold increase of GlcCer content in LCLs derived from patients compared to controls, with the C_16_ lipid being the most abundant GlcCer species. Moreover, we showed that there is an apparent compensatory effect between NLGase and the lysosomal glucosylceramidase (GCase), since we found that the activity of GCase was three-fold higher in LCLs derived from patients compared to controls. We conclude that the c.1780G>C mutation results in NLGase loss of function with abolishment of the enzymatic activity and accumulation of GlcCer accompanied by a compensatory increase in GCase.

## 1. Introduction

Sphingolipids (SLs) are a class of lipids mainly associated with the external leaflet of the plasma membrane (PM) of all eukaryotic cells, playing an important role in the structural integrity of the PM and cellular signaling [1]. Glycosphingolipids (GSLs) are SLs with a head-group formed by a mono- or oligosaccharide moiety. Glucosylceramide (GlcCer) is the simplest member of GSLs and it is formed in the Golgi complex by the glycosylation of ceramide [2]. SLs play a fundamental role in cell physiology and this can be demonstrated by the numerous genetic diseases that arise from mutations in enzymes involved in SL metabolism and transport [3,4]. Cells can alter their lipid composition by the action of different hydrolases that are active at the lysosomes or at the PM, such as sphingomyelinase (SMase), β-hexosaminidase (β-Hex), β-galactosidase (β-gal), β-glucocerebrosidase (GCase), and the non-lysosomal β-glucosylceramidase (NLGase) [5,6].

GCase and NLGase are both involved in the catabolism of GlcCer to ceramide and glucose. GCase (EC 3.2.1.45) is encoded by the *GBA* gene (MIM_606463), located on chromosome 1q21. GCase is a membrane glycoprotein of 497 amino acids with a β-barrel structure, ubiquitously expressed in all tissues and mainly localized in lysosomes. The catalytic site of GCase contains two highly conserved residues of glutamic acid, which are necessary for the two-step mechanism of action, the nucleophilic attack, and the subsequent protonation. Loss of function mutations in the *GBA* gene cause Gaucher disease, the most common lysosomal storage disorder [7,8].

NLGase (EC 3.2.1.45) is encoded by the *GBA2* gene localized on chromosome 9p13.3. The catalytic site of the protein is characterized by both nucleophile and acid/base residues, Glu-528 and Asp-678 respectively. These residues define NLGase as a retaining β-glucosidase belonging to the CAZy glycosyl hydrolase family 116 (GH116) [9]. As previously described, the retaining β-glucosidases utilize a double-displacement mechanism [10]. In the first step of the reaction, called glycosylation, the nucleophile residue attacks the glucose anomeric center to create the glycosyl-enzyme intermediate, whereas the acid/base residue protonates the glycosydic oxygen, leading to the release of a glycone [11]. In the second step, the group working as an acid in the first step acts as a base catalyst that together with incoming water determines the de-glycosylation of the nucleophile. This mechanism allows the retention of the configuration at the anomeric carbon of the released glucose molecule [12].

The intracellular localization of NLGase has been controversial. It was reported to be a single pass transmembrane protein [13] and later was identified as a cytoplasmic membrane-associated protein of the ER and Golgi complex [14]. The activity of NLGase at the PM is directly modulated by the efflux of protons through the proton pumps associated with the cell surface [15,16]. 

The pathological involvement of NLGase was initially studied by generating *GBA2*-knockout mice [17]. Knockout mice present impairment in liver regeneration [18], and male infertility due to GlcCer accumulation. This accumulation causes dysregulation of lipid homeostasis due to a more ordered lipid composition of the PM [19,20]. As a result, the cytoskeletal dynamics are altered and the formation of sperm-head shaping and acrosome is affected [19].

Despite the abnormal GlcCer accumulation in brain, *GBA2*-knockout mice do not display any neurological symptoms or defects [19]. On the contrary, *GBA2*-knockdown zebrafish show abnormal motor neuron development [21], and mutations in the human *GBA2* gene have been found to lead to neurological disorders like spastic ataxia (SA) [22,23], hereditary spastic paraplegia (HSP) [21,24,25], and more recently Marinesco-Sjogren-Like Syndrome [26]. The molecular mechanism(s) leading to the development of disease are not currently known. Only one mutation of *GBA2* has been so far functionally characterized in vivo (i.e., zebrafish model) [21]. A more detailed biochemical analysis of the different mutants of *GBA2* in patient derived cells is still missing.

We have previously identified a *GBA2* missense mutation [c.1780G>C (p.Asp594His)] in a Cypriot family with progressive spastic ataxia [22]. In vitro characterization of this mutation in COS7 and HeLa cells showed that it causes a reduction at both the protein and enzyme activity levels [25]. In the present study we have undertaken the biochemical characterization of the *GBA2* c.1780G>C missense mutation in lymphoblastoid cell lines (LCLs) derived from spastic ataxia patients homozygous for the mutation. Our results contribute to the understanding of the biochemical consequences of mutations in the *GBA2* gene.

## 2. Results

### 2.1. The c.1780G>C Mutation Results in NLGase Loss of Activity and GlcCer Accumulation

**Figure 1:**
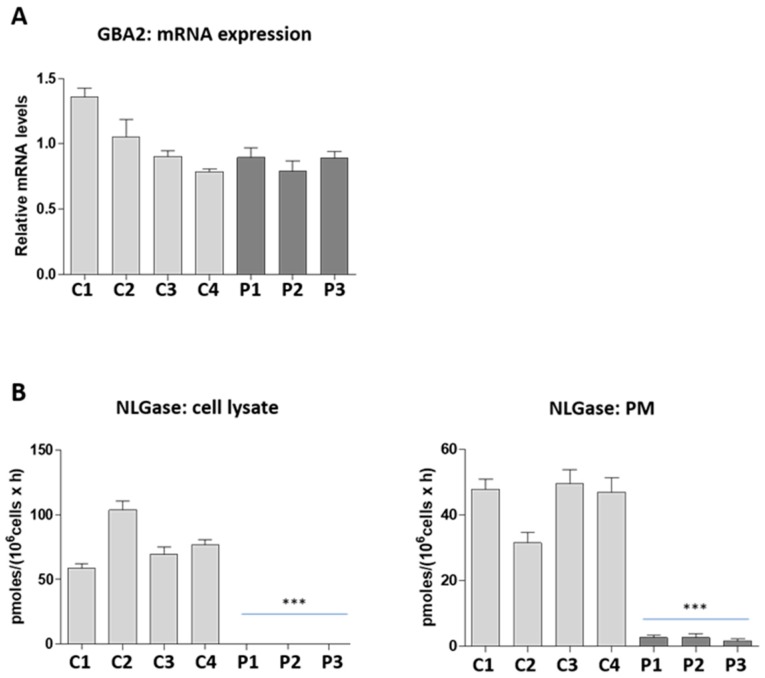
*GBA2* mRNA expression and non-lysosomal glucosylceramidase (NLGase) activity. (**A**) The graph represents the *GBA2* mRNA levels of controls (light grey) and patients (dark grey) relative to the average value of controls (*n* = 4) after normalization with the endogenous β-actin gene (*ACTB*). Values represent the mean ± SEM of two independent triplicate experiments. (**B**) NLGase activity associated with the total cell lysates and plasma membrane (PM) of controls (light grey) and patients (dark grey) derived lymphoblastoid cell lines (LCLs). Enzymatic activity was expressed as pmoles/10^6^ cells/h. Data are expressed as mean ± SD of three independent triplicate experiments (*** *p* < 0.0001 vs. controls).

*GBA2* mRNA expression levels were measured in LCLs obtained from four healthy individuals and from three patients homozygous for the c.1780G>C mutation. As shown in Figure 1-panel A, the same level of *GBA2* transcript was found in control and patient LCLs, suggesting that the mutation does not affect the *GBA2* mRNA expression and stability.

Subsequently, we measured the NLGase enzymatic activity on the total cell lysate of the same LCLs. We observed that NLGase activity was almost undetectable in patients’ cells with respect to controls (Figure 1B), which are characterized by an average specific activity of 78 ± 19 pmoles/10^6^ cells/h.

In several cell lines of different origin, NLGase was found to be associated with the external leaflet of the PM where it catalyzes the in situ hydrolysis of glucosylceramide (GlcCer) to ceramide [27,28,29]. For this reason, we measured the enzymatic activity of NLGase directly at the cell surface of control and patient derived lymphoblastoid living cells. In patients’ cells NLGase activity was also strongly reduced at the cell surface, showing 2–4% of residual activity with respect to that found in controls (Figure 1B; controls 44 ± 8 pmoles/10^6^ cells/h, patients 2.3 ± 0.5 pmoles/10^6^ cells/h). We can exclude that the enzymatic activity measured at the PM was due to other β-glucocerebrosidases because, by adding AMP-DNM, a specific inhibitor of NLGase to the assay solution abolished the enzymatic activity at the cell surface. Our data demonstrate that the presence of the homozygous *GBA2* c.1780G>C mutation results in an important loss of NLGase activity. 

Furthermore, we assessed the effect of the NLGase loss of function on the GlcCer content. To this purpose, total lipid extracts obtained from the same LCLs used for the evaluation of the enzymatic activity were subjected to SFC-MS/MS analysis. This method is capable of quantifying and also distinguishing GlcCer from galactosylceramide (GalCer).

As shown in Table 1, patient derived LCLs are characterized by a two-fold increase in the GlcCer content with respect to controls. No differences were found among controls and cells expressing the mutated protein in the level of GalCer. Moreover, no difference was found in cellular cholesterol content between patients and controls, suggesting that GBA2 loss-of-function does not affect cholesterol homeostasis. In Gaucher disease, where we have GCase loss-of-function, the accumulated GlcCer is converted to glucosphingosine (GlcShp) by the action of the acid ceramidase [30]. We therefore also analyzed the levels of GlcShp by SFC-MS/MS and these were found to be hardly detectable, without any significant difference between control and patient cells. 

Quantitative analysis of the different molecular species of GlcCer showed that C16 is the most abundant species in both control and patient derived LCLs. In addition, it emerged that all the molecular species are doubled in pathological cells with respect to controls (Table 2).

**Figure 2:**
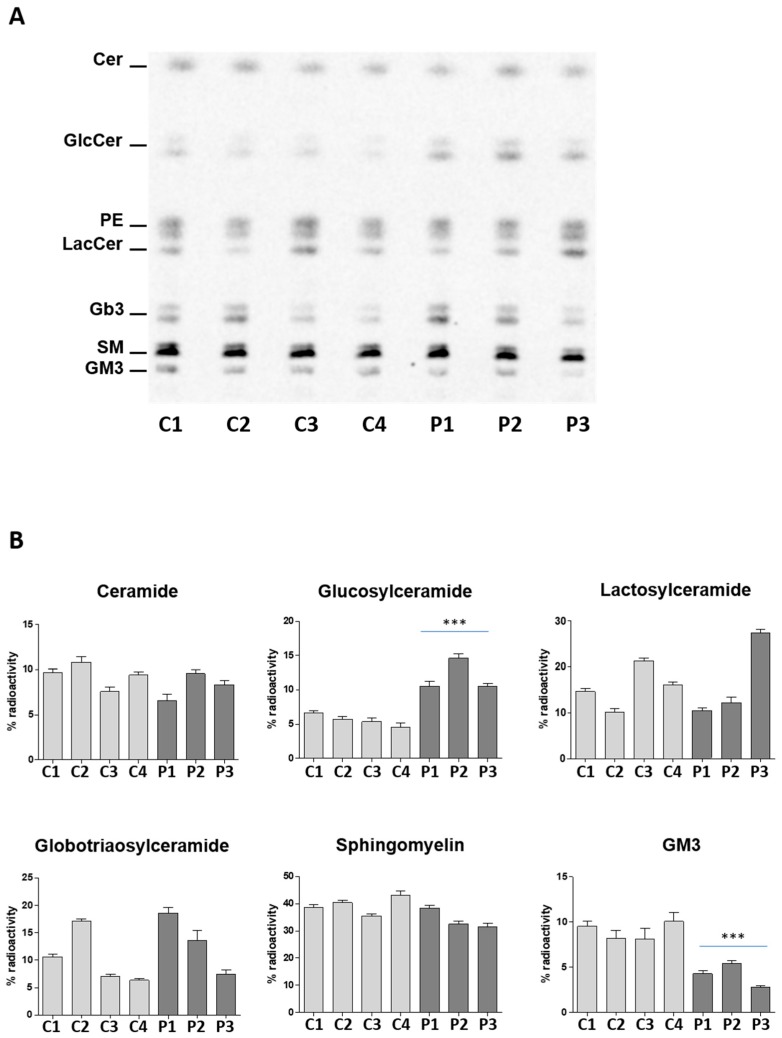
Radioactive sphingolipid pattern of control and patient LCLs. Total lipid extracts were separated by thin layer chromatography using the solvent system Chloroform/Methanol/Water 110:40:6 (*v*:*v*:*v*). (**A**) Representative digital autoradiogram obtained by the Beta-Imager ^T^Racer equipment (BioSpace Lab). Same quantities of radioactivity were applied per lane. Ceramide (Cer), glucosylceramide (GlcCer), phosphatidylethanolamine (PE), lactosylceramide (LacCer), globotriaosylceramide (Gb3), sphingomyelin (SM), and ganglioside GM3. (**B**) Distribution of the radioactive sphingolipids associated with the total lipid extract expressed as % of the total radioactivity. Data are expressed as mean ± SD of three independent triplicate experiments (*** *p* < 0.0003 vs. controls).

In order to show that the increase of GlcCer was the result of an impairment of its catabolism, we labeled the cell sphingolipids at steady state using radioactive sphingosine [1-^3^H]Sph. This experimental procedure is based on the recycling of the radioactive precursor [1-^3^H]Sph that is used by the anabolic pathways as the endogenous counterpart. When there is an impairment of a catabolic enzyme (i.e., NLGase), the [1-^3^H]Sph is not further recycled and no degraded radioactive sphingolipid accumulates. As shown in Figure 2, despite the inter-individual variability in the content of lactosylceramide (LacCer) and globotriaosylceramide (Gb3), no significant differences were found between controls and patients for the other sphingolipids characteristic of LCLs. The only exception was the GlcCer and the ganglioside GM3 content. All patient derived LCLs showed an increase of more than two-fold of radioactive GlcCer with respect to control LCLs as well as a decrease of the radioactive GM3. The reduction in patient LCLs of the ganglioside GM3, which is a lipid typically associated with the external leaflet of the cell PM, with the concomitant increase in GlcCer, supports the hypothesis that the impairment of the catabolic pathway occurs at the cell PM level.

### 2.2. GCase Activity is Up-Regulated in GBA2-Deficient LCLs Particularly at the PM

**Figure 3:**
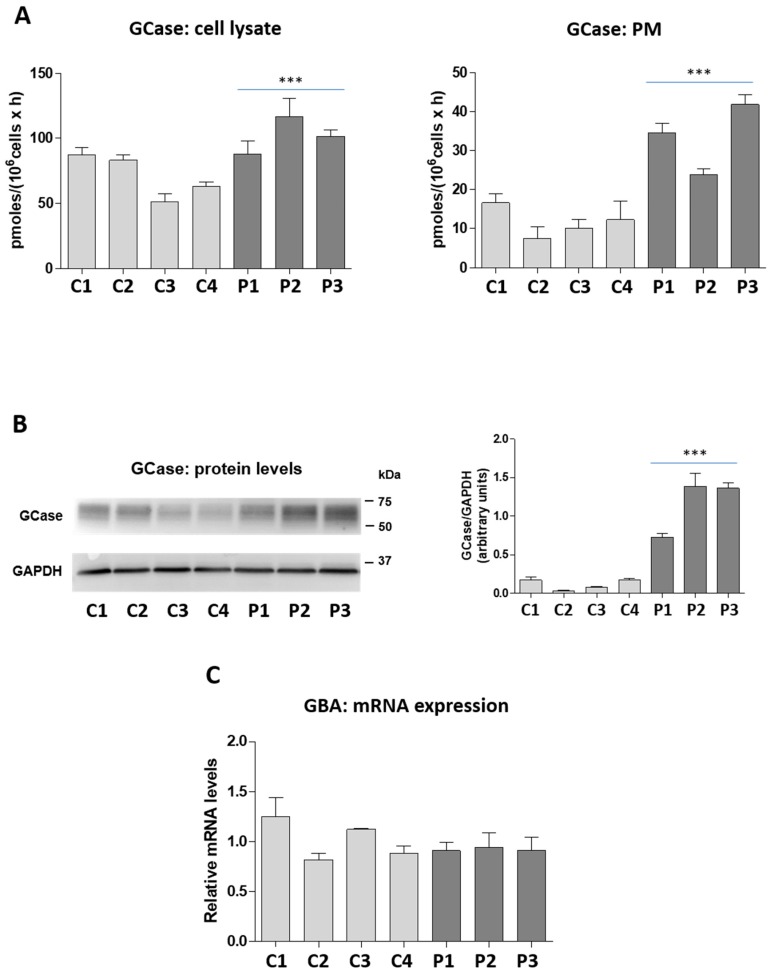
Activity and protein levels of β-glucocerebrosidase (GCase) and *GBA* mRNA expression. (**A**) GCase activity associated with the total cell lysate and plasma membrane of control (light grey) and patient (dark grey) derived LCLs. Activities were expressed as pmoles/10^6^ cells/h. Data are expressed as mean ± SD (*n* = 4, *** *p* < 0.0001 vs. controls). (**B**) Immunoblot of GCase and control GAPDH accompanied by the semi-quantitative graph of normalized GCase/GAPDH. Data are expressed as mean ± SD (*n* = 4 *** *p* < 0.0001 vs. controls). (**C**) The graph represents the *GBA* mRNA levels of controls (light grey) and patients (dark grey) relative to the average value of controls (*n* = 4), after normalization with the endogenous β-actin gene (*ACTB*). Values represent the mean ± SEM of two independent triplicate experiments.

Several lines of evidence suggest the existence of a cross-talk among the enzymes involved in sphingolipid (SL) catabolism, since modification in the activity/expression of one enzyme could affect that of others [31]. Besides NLGase, another important enzyme involved in GlcCer catabolism is β-glucocerebrosidase (GCase) encoded by the *GBA* gene. Unlike NLGase, GCase is mainly a lysosomal enzyme and only partially associated with the external leaflet of the PM [15]. To investigate the possibility of a cross-talk between GCase and NLGase, we measured the activity of GCase both intracellularly and at the cell surface in control and patient derived LCLs. As shown in Figure 3–panel A, GCase activity was increased in total cell lysates derived from patients’ cells with respect to controls. The average activity of GCase was 102 ± 14 pmoles/10^6^ cells/h and 71 ± 17 pmoles/10^6^ cells/h in patient and control derived LCLs, respectively. A marked increase, about three-fold, of GCase activity was also observed at the PM level in patients compared to control LCLs (patients: 33 ± 9 pmoles/10^6^ cells/h; control: 11 ± 4 pmoles/10^6^ cells/h). Interestingly, the augmented enzymatic activity is associated with an increase in the GCase protein levels but not in *GBA* mRNA expression (Figure 3B,C).

A substantial body of evidence shows the presence and action of mature and active lysosomal enzymes at the PM in addition to the lysosomes [15,32]. We decided to investigate the activity of two main hydrolases involved in SL catabolism, β-galactosidase and β-hexosaminidase, both intracellularly and at the PM level. We observed an increase only of the β-galactosidase activity associated with the cell surface of LCLs obtained from patients with respect to controls (Table 3).

## 3. Discussion

Several lines of evidence indicate that the regulation of GlcCer levels is important for cell homeostasis. GlcCer is a minor component of almost all membranes of eukaryotic cells suggesting an evolutionary strategy aimed to limit its presence. Indeed, de novo biosynthesized GlcCer is mainly used as a building block for the biosynthesis of complex GSLs [33]. 

In mammalian cells, GSL catabolism occurs by the sequential hydrolysis of the saccharidic chain by removing the reducing sugar. Lysosomes are involved in the catabolism of the endocytic portion of the cell PM and could be considered the principal site, together with the endoplasmic reticulum and Golgi complex, responsible for GSLs turnover [34]. On the other hand, the fine tuning of the GSLs composition is triggered directly at the cell PM by the action of specific glycohydrolases. In particular, the same enzymes that are associated with the lysosomes, such as sialidase Neu1, beta-hexosaminidase, beta-galactosiadase, and GCase, are present at the cell surface, even if in very small amounts, along with the sialidase Neu3 and NLGase, which are enzymes primarily residing at the PM [5]. Loss of function mutations in the lysosomal glycohydrolases determine the onset of lysosomal storage disorders, characterized by the accumulation of non-catabolized substrates. 

NLGase is currently the most studied enzyme among the PM glycohydrolases. NLGase deficient mice, obtained both, by gene knockout and by pharmacological inhibition of the enzyme, showed an abnormal GlcCer accumulation in multiple tissues, including brain, liver, and testis. These data were quite surprising considering that the large amount of the GSL catabolism occurs in lysosomes. In addition, these mice were characterized by impaired liver regeneration and male infertility but no neurological involvement was observed [17,18,19]. However, in humans *GBA2* gene mutations are found in spastic ataxia and spastic paraplegia patients (SPastic Gait locus #46, SPG46) [23,24,25,35]. Among them, a c.1780G>C (p.Asp594His) missense mutation located in exon 11 of the *GBA2* gene was identified in a Cypriot consanguineous spastic ataxia family [22]. 

We used LCLs obtained from three patients of this family, who are homozygous for the *GBA2* c.1780G>C missense mutation, to evaluate the effect of the mutation on NLGase activity. By an in vitro enzymatic assay based on the use of CBE to block GCase activity, we found that the mutation strongly affects NLGase activity. Indeed, in pathological cells we were not able to detect any NLGase activity in the total cell lysate, and only a very low residual activity at the cell surface when compared with LCLs derived from four healthy controls. Similar to the results observed in mice, we found that the NLGase loss of function in patient derived LCLs is responsible for an increase of GlcCer content that reaches two-fold to that found in control cells. In addition, patient LCLs show an increased activity of GCase with respect to that measured in controls. In particular, GCase activity associated with the PM of patient derived LCLs is three-fold higher than that of control LCLs. This presumably compensatory effect has already been described in fibroblasts derived from patients affected by Gaucher disease, where the GCase loss of function induced an increase in NLGase activity [31]. Indeed, by the evaluation of the total cell β-glucocerebrosidase activity we did not find statistically significant differences between control and patient derived LCLs (Figure S1). 

Taken together, the data, herein reported, further support an important role of NLGase in GlcCer metabolism and the existence of a cross-talk among the enzymes involved in GSL catabolism. Despite this new evidence, the challenges for future studies remain: (i) to explain why NLGase loss of function with concomitant increase of GCase could result in GlcCer accumulation and (ii) which are the molecular mechanisms linking the NLGase-dependent GlcCer accumulation with the onset of spastic ataxia.

## 4. Materials and Methods

### 4.1. Cell Culture

Seven LCLs (3 patients and 4 controls) were available, which were expanded and sub-cultured for the purposes of this study. Cells were grown in culture medium RPMI (Roswell Park Memorial Institute medium) supplemented with 10% FBS (fetal bovine serum), 1% penicillin/streptomycin and 1% glutamine, and expanded for a period of 6 weeks at 37 °C in a 5% CO_2_ incubator. Genotypes of all patient derived LCLs sub-cultures were reconfirmed with Sanger sequencing and healthy controls were confirmed as homozygous wild-type.

All subjects gave their informed consent for inclusion before they participated in the study. The study was conducted in accordance with the Declaration of Helsinki, and approved by the National Bioethics Committee of Cyprus (ΕΕΒΚ/EΠ/2013/28, date of approval 14 May 2015).

### 4.2. Evaluation of Enzymatic Activities in Cell Lysates

The enzymatic activities associated with total cell lysates were determined by an assay based on the use of fluorogenic substrates as previously described [36]. To evaluate NLGase activity, cell lysates were pre-incubated for 30 min at room temperature in McIlvaine buffer (pH 6) with 1 mM CBE (Conduritol-B-epoxide, Merck, Darmstadt, Germany), a specific inhibitor of GCase [37]. For the measurement of GCase activity, cell lysates were pre-incubated for 30 min at room temperature in McIlvaine buffer (pH 5.2) containing 0.1% Triton X100(Merck, Darmstadt, Germany) with 5 nM AMP-dNM (adamantane-pentyl-dNM; *N*-(5-adamantane-1-yl-methoxy-pentyl) deoxynojirimycin, (A generous gift from Prof. Aerts JM form Leiden University) a specific inhibitor of NLGase. The total β-glucocerebrosidase assay was performed using the same procedure without inhibitors and detergents. At the end of the pre-incubation, the reactions were started by the addition of 25 µL of 4-Methylumbelliferyl β-d-glucopyranoside (MUB-β-Glc, Glycosynth, Warrington, UK) at a final concentration of 6 mM. To measure β-galactosidase and β-hexosaminidase activities, the fluorogenic substrates used were 4-Methylumbelliferyl β-d-galactopyranoside (MUB-β-Gal) and 4-Methylumbelliferyl *N*-acetyl-β-d-glucuronide (MUG) (all from Glycosynth, Warrington, UK), respectively. Aliquots of cell lysates were incubated with 25 µL of McIlvaine buffer 4× (0.4 M citric acid /0.8 M Na_2_HPO_4_) pH 5.2 and the specific fluorogenic substrates at a final concentration of 500 µM. Water was added to reach the final volumes of 100 µL. At different time points the reaction was stopped by adding 9 volumes of 0.25 M glycine pH 10.7 (Sigma-Aldrich, St. Louis, MO, USA). The fluorescence was detected by a Victor microplate reader (Perkin Elmer, Waltham, MA, USA). Standards of free 4-methylumbelliferone (MUB) were used to construct calibration curves. The enzymatic activities were expressed as pmoles of product/10^6^ cells /h.

### 4.3. Evaluation of Enzymatic Activities at the Cell Surface of Living Cells

PM-associated activities of total β-glucocerebrosidase, GCase, NLGase, β-galactosidase and β-hexosaminidase were assessed in living cells, plated in a 96-well microplate at a density of 200,000 cells/well, by a high throughput live cell-based assay as previously described [15,27,31,36]. To distinguish between GCase and NLGase activities, cells were pre-incubated for 30 min at room temperature in DMEM-F12 without phenol red (Thermo Fisher Scientific, Waltham, MA, USA) containing 5 nM AMP-DNM or 1 mM CBE, respectively [38]. Total β-glucocerebrosidase assay was performed using the same procedure without any inhibitors. Activities were assayed using the artificial substrate MUB-β-Gal for β-galactosidase, MUG for β-hexosaminidase, and MUB-β-Glc for β-glucocerebrosidases GCase and NLGase. The fluorogenic substrates were solubilized in DMEM-F12 without phenol red at pH 6, with final concentrations of 250 µM, 1 mM, and 6 mM, respectively. Aliquots of medium (10 µL) were analyzed at different time points by a Victor microplate reader (Perkin Elmer, Waltham, MA, USA), after adding 190 µL of 0.25 M glycine, with a pH of 10.7. Standards of free MUB were used to construct calibration curves. The enzymatic activities were expressed as pmoles of product/10^6^ cells/h. The experimental design included internal controls. In particular, this method is based on the observation that the fluorogenic substrates commonly used for the in vitro assay of glycohydrolytic activities are not taken up by living cells. To assess that the substrate hydrolysis occurs only upon the activity of PM enzymes, a series of controls was performed. In the used experimental conditions, we did not observe any intracellular fluorescence, evaluated by both fluorescent microscopy and fluorimetric analysis of the cells lysed in 0.25 M glycine (pH 10.7), indicating that the substrates were not able to cross the cell membrane. Moreover, we verified that the artificial substrates did not undergo either spontaneous or secreted enzyme-driven hydrolysis by the establishment of an appropriate control without cells or by the solubilization of MUB-substrates directly in cell culture medium in the presence or not of cells.

### 4.4. Real-Time PCR

Total RNA was isolated from the LCLs using the RNeasy^®^ Midi kit (Qiagen, Hilden, Germany) as described in the manufacturer’s instructions. cDNA synthesis was performed using 1μg of total RNA according to the instructions of the Protoscript^®^ M-MuLV II First Strand cDNA Synthesis Kit (New England Biolabs, Ipswich, Massachusetts, USA). Real-time PCR was carried out using the CFX96 Real-Time system (Bio-Rad, Hercules, CA, USA) and the amplification was done using the SsoFast EvaGreen Supermix according to the manufacturer’s instructions (Bio-Rad, Hercules, CA, USA). The sequences of the primers are available upon request. *GBA1* and *GBA2* mRNA expression levels were normalized to the actin house-keeping gene (*ACTB*) and relative mRNA expression was calculated according to the ΔΔCT method.

### 4.5. Immunoblotting

Equivalent amounts of proteins associated with total cell lysates, determined by DC Protein Assay (Bio-Rad, Hercules, CA, USA), were separated on polyacrylamide gels and then transferred to PVDF (Polyvinylidene fluoride) membranes by electroblotting [29]. Blots were incubated with monoclonal rabbit anti-GCase (ab128879, Abcam, Cambridge, UK) or polyclonal rabbit anti-GAPDH (G9545, Sigma-Aldrich) primary antibodies at 4 °C overnight, followed by incubation with goat anti-rabbit HRP-conjugated (7074, Cell Signaling) secondary antibody and detection with a chemiluminescent kit (WESTAR ηC, Cyanagen, Bologna, Italy). Digital images were obtained by the chemiluminescence system Alliance Mini HD9 (UVItec, Cambridge, UK).

### 4.6. Cell Sphingolipid Labelling with [1-^3^H]-Sphingosine

[1-^3^H]-sphingosine was administered as tracer in non-bioactive concentration for 2 h (pulse) followed to 96 h (chase), to allow steady state metabolic labelling of all cell SLs [39]. Briefly, [1-^3^H]-sphingosine dissolved in methanol was transferred into a sterile glass tube, dried under a nitrogen stream and then solubilized in an appropriate volume of pre-warmed (37 °C) cell culture medium to obtain a final concentration of 30 nM. The correct solubilization was verified by measuring the radioactivity associated with an aliquot of the medium using a β-counter (PerkinElmer, Waltham, MA, USA). After 2 h of incubation (pulse) the medium was removed, and the cells were incubated for 96 h (chase) in fresh culture medium without radioactive sphingosine. After chase, cells were collected, lyophilized and subjected to lipid extraction and SLs analysis. Total lipids from lyophilized cells were extracted with chloroform:methanol:water 20:10:1 by volume, followed by a second extraction with chloroform:methanol: 2:1 by volume. The radioactivity associated with total lipid extract, was evaluated by liquid scintillation, using a β-counter system (PerkinElmer).

[^3^H]SLs of total extracts were separated by high performance thin layer chromatography (HPTLC), using the solvent system chloroform:methanol:water 110:40:6 by volume. [^3^H]SLs were identified by digital autoradiography using ^T^Racer system (Biospace Lab) and quantified with M3vision software. The lipid identification was performed using purified radioactive standards.

### 4.7. Lipid Analysis by SFC-MS/MS

Quantitative analysis of glucosylceramide, galactosylceramide, glucosylsphingosine and galactosylsphingosine was performed by Lipidomics Shared Resources Analytical Unit (Medical University of South Carolina, Charleston, SC, USA) [40]. Briefly, quantitative analysis of sphingolipids is based on eight-point calibration curves generated for each target analyte. The synthetic standards, along with a set of internal standards, were spiked into an artificial matrix and subjected to an identical extraction procedure as the biological samples. These extracted standards were then analyzed by the SFC-MS/MS system operating in positive MRM mode employing a gradient elution. Peaks for the target analytes and internal standards were recorded and processed using the instrument’s software system. The calibration curve for a particular analyte was generated by plotting the analyte/internal standard peak area ratio against analyte concentrations. Any sphingolipid for which no standards were available was quantitated using the calibration curve of its closest counterpart. Separation of galactosylceramide and glucosylceramide was performed by SFC-MS/MS. The equipment consisted of a Waters UPC^2^ system coupled to a Thermo Scientific Quantum Access Max triple quadrupole mass spectrometer, equipped with an ESI (electrospray ionization) probe operating in the multiple reaction monitoring positive ion mode tuned and optimized for the Waters UPC^2^ system. Chromatographic separations were obtained utilizing carbon dioxide gas and 1 mM ammonium formate in 0.2% formic acid in the methanol mobile phase.

Analytical results were expressed as pmoles of lipid/mg of total cellular proteins. Data were the mean of two independent triplicate experiments.

## Figures and Tables

**Table 1:** Hexosylceramides of lymphoblastoid cell lines (LCLs) from controls and patients. Glucosylceramide (GlcCer) and galactosylceramide (GalCer) contents were evaluated by SFC-MS/MS in controls (WT, *n* = 4) and patients (c.1780G>C, *n* = 3) LCLs. Data are expressed as pmoles/mg of cell protein ± Error (*n* = 3).

Hexosylceramides of LCLs from Controls and Patients (Pmoles/mg Cell Proteins)		
	WT	c.1780 G>C
Glucosylceramide	1080 ± 107	2009 ± 114
Galactosylceramide	9 ± 3	6 ± 2

**Table 2:** SFC-MS/MS analysis of glucosylceramide (GlcCer) molecular species in control (WT, *n* = 4) and patient (c.1780G>C, *n* = 3) derived LCLs. Data are the mean of three independent experiments and are expressed as pmoles/mg of cell proteins ± SEM.

Glucosylceramide Molecular Species of LCLs from Controls and Patients (Pmoles/mg Cell Proteins)												
		C14	C16	C18	C18:1	C20	C22	C22:1	C24	C24:1	C26	C26:1
**GlcCer**	*WT*	13 ± 2	611 ± 100	24 ± 5	1 ± 0.1	28 ± 7	76 ± 14	6 ± 1	143 ± 22	150 ± 21	9 ± 1	15 ± 2
	*c.1780 G>C*	21 ± 1	1139 ± 103	51 ± 2	1.3 ± 0.2	63 ± 7	145±7	11 ± 1	261 ± 20	278 ± 44	15 ± 2	21 ± 1

**Table 3:** Enzymatic activities associated with total cell lysate and the plasma membrane (PM) of control (WT) and patient (c.1780 G>C) derived LCLs. Enzymatic activities were expressed as pmoles/10^6^ cells/h ± error.

Enzymes	Cell Lysate		PM	
	WT	c.1780 G>C	WT	c.1780 G>C
β-Galactosidase	1284 ± 246	1399 ± 149	14 ± 4	30 ± 4
β-Hexosaminidase	1868 ± 233	1961 ± 206	24 ± 6	35 ± 15

## Supplementary Materials

Supplementary materials can be found at http://www.mdpi.com/1422-0067/19/10/3099/s1.
