# Supplementary material for: Biochemical Characterization of the GBA2 c.1780G>C Missense Mutation in Lymphoblastoid Cells from Patients with Spastic Ataxia
Source: Int J Mol Sci. 2018 Oct 10;19(10):3099. doi: 10.3390/ijms19103099 (PMC6213336; doi:10.3390/ijms19103099)
Supplement: Supplementary file 1 [file ijms-19-03099-s001.pdf]

Supplementary Figure 1

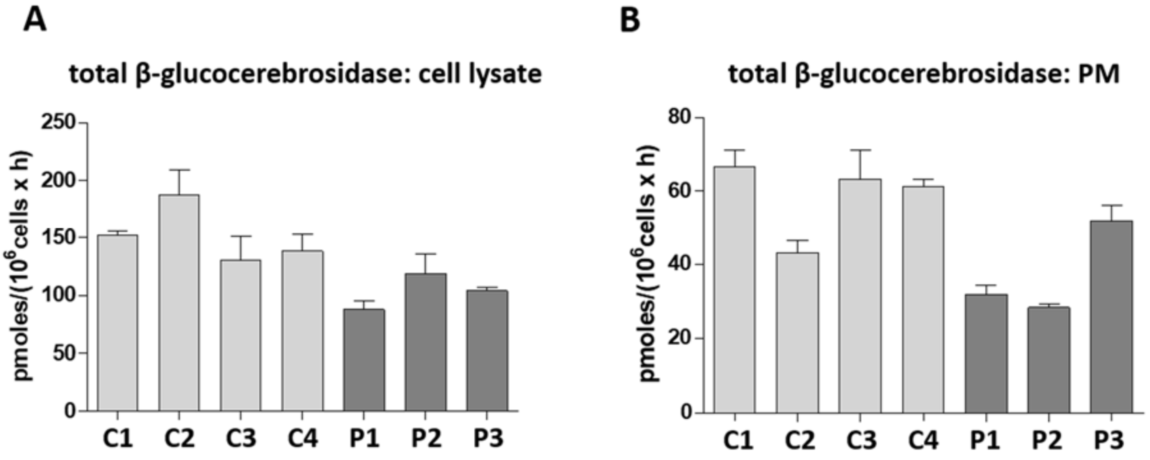

Figure S1. Total β-glucocerebrosidase activity A) Total β-glucocerebrosidase activity associated with the total cell lysates and B) of plasma membrane (PM) of controls (light grey) and patients derived LCLs (dark grey). Enzymatic activity was expressed as pmoles/10^6 cells/h. Data are expressed as mean±SD of three independent triplicate experiments.
